# Delayed Presentation of Foot Compartment Syndrome

**DOI:** 10.7759/cureus.8628

**Published:** 2020-06-15

**Authors:** Anupam K Gupta, Monica I Burgos, Miguel Lopez-Viego

**Affiliations:** 1 Minimally Invasive Surgery, University of Miami Hospital, Miami, USA; 2 Internal Medicine, Universidad Autonoma de Guadalajara, Guadalajara, MEX; 3 Surgery, Charles E. Schmidt College of Medicine, Florida Atlantic University, Boca Raton, USA; 4 Surgery, Bethesda Hospital, Boynton Beach, USA

**Keywords:** compartment syndrome, foot compartment syndrome, opiod abuse, compartment syndrome leg, fasciotomy

## Abstract

A 30-year-old male after consuming alcohol and drugs lost consciousness and passed out in his bathroom. After 10 hours, he was found immobilized stuporous on his bathroom floor and brought by emergency service to the emergency room. He was found to have compartment syndrome of the right lower limb and underwent emergency fasciotomy of three compartments of the thigh and four compartments of the leg. The postoperative course was complicated by acute kidney injury necessitating hemodialysis. On postoperative day three from the index fasciotomy patient was found to develop foot compartment syndrome despite not having foot compartment involved in the initial presentation. In opioid abuse, patients’ additional compartments can get involved during the disease process, and it is essential to identify them with regular physical examination and use of biomarkers.

## Introduction

Compartment syndrome is an acute surgical emergency, with one common etiology being prolonged immobilization secondary to overconsumption of opioids [1]. These patients provide a unique subset of management challenges. In these patients relief of pain, creatinine phosphokinase downtrends, and decreasing swelling can be misleading about the adequacy of fasciotomy in the postoperative recovery. Early recognition of identifying all muscle groups is the goal for these patients to provide adequate decompressive fasciotomy. A multidisciplinary approach is needed for the management of pain, renal injury, and strict vigilance for the development of uncommon compartments syndrome [2].

## Case presentation

A 30-year-old male patient with no significant past medical history presented to the emergency room in a confused state after being found immobile near the staircase. He was confused and unable to move his right lower limb and only remembered consuming alcohol and drugs the night before. At the arrival time in the emergency room, the patient had a heart rate of 100 beats per minute, sinus rhythm, tachypnea (respiratory rate to 24 per minute), blood pressure of 100/61 mmHg, afebrile and saturating 95% on room air. His physical examination was remarkable for the inability to move his right lower limb with paresthesia at the right lower leg. At the time, the patient had an excellent palpable pulse in all four extremities. A Foley catheter was placed in the emergency room as the patient could not ambulate to the restroom, and it showed dark tea-colored urine. Complete blood revealed elevated white blood cell count to 20.3k/ml, hemoglobin of 19.1 grams/deciliter. Remaining blood work was significant for high potassium to 5 mmol/L, blood urea nitrogen to 35 mg/dl and creatinine to 3 mg/dL, creatinine phosphokinase levels more than 4100 IU/L. Toxicology revealed alcohol, benzodiazepine, and opiates in urine. Urine was dark tea-colored, and the patient had rhabdomyolysis with myoglobinuria. 

**Figure 1:**
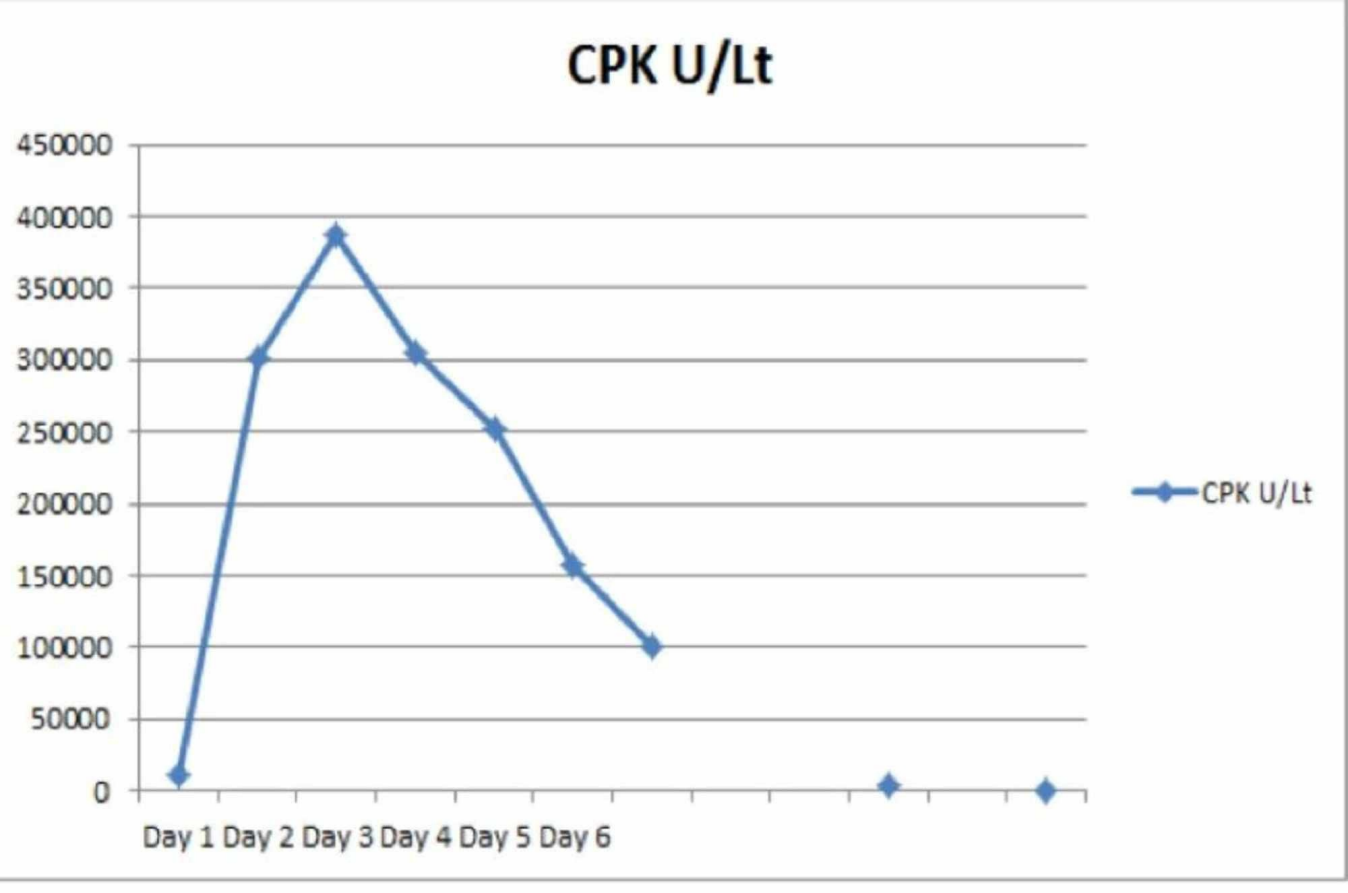
Creatine phosphokinase trends over various days. Over the subsequent days by day 11 levels reached base line CPK, creatine phosphokinase

Because of acute right lower limb compartment syndrome, the patient underwent emergency three-compartment fasciotomy of the right thigh and four compartments fasciotomy of the right lower limb. Adequate fasciotomy with generous incisions on either side of the leg and thigh was done. Hemodialysis was initiated on postoperative day 1 because of hyperkalemia (6.8 mmol/L). Over the subsequent days, creatinine phosphokinase was trended, and the down-trending level was considered suggestive of adequate fasciotomy (Figure 1).

The patient had persistent post-surgical swelling due to lymphedema and acute kidney injury-causing fluid retention. Lymphedema was secondary to disruptions of lymphatics due to extensive fasciotomy.

**Figure 2:**
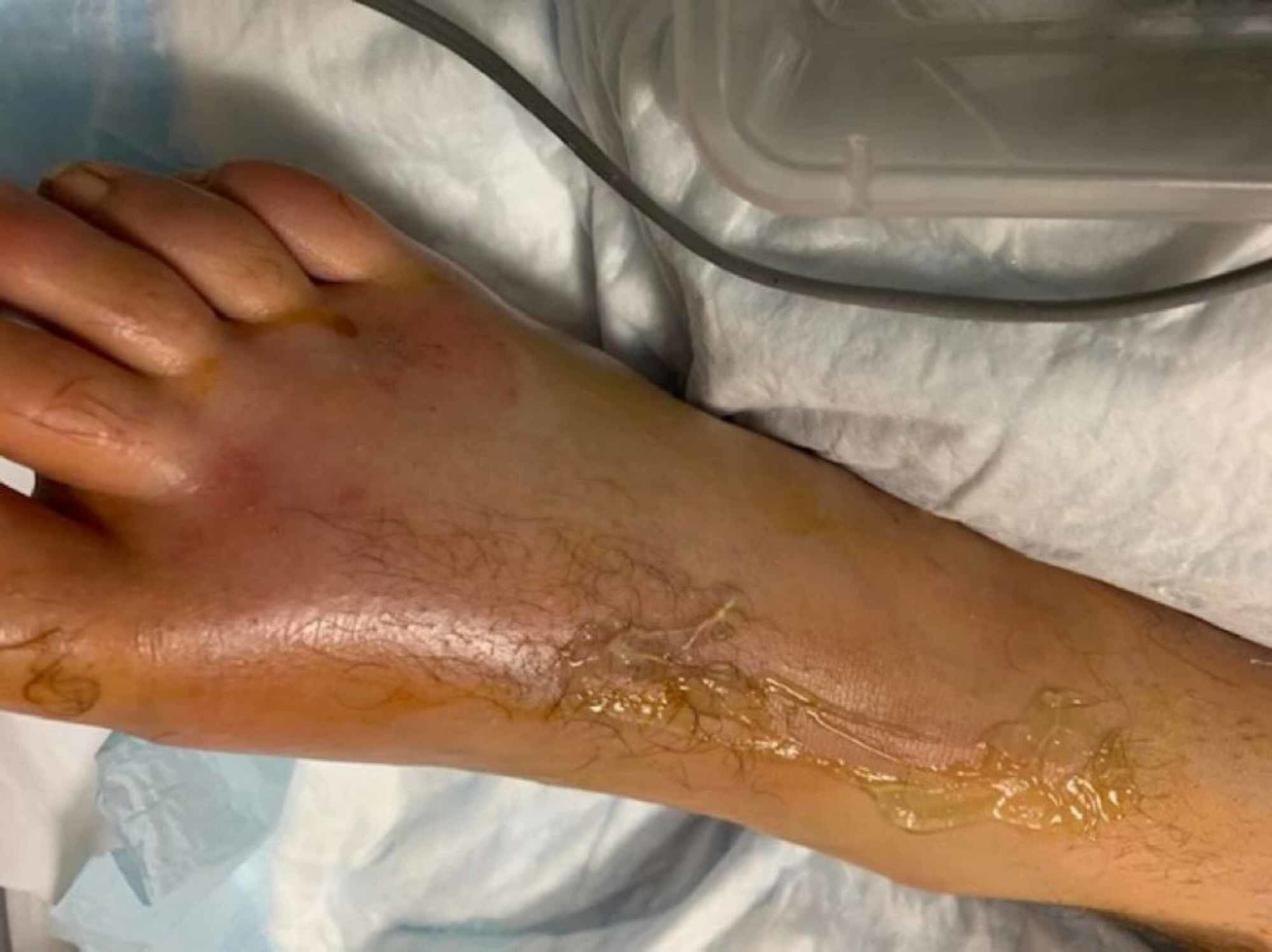
Swollen foot with a lack of pulse

Because of the patient's prior narcotic abuse, the patient needed higher doses of opiates postoperatively, and it was challenging to evaluate pain as a symptom to assess the recovery of his right lower limb. On postoperative day 3, his foot showed swelling with a lack of pulse (Figure 2).

**Figure 3:**
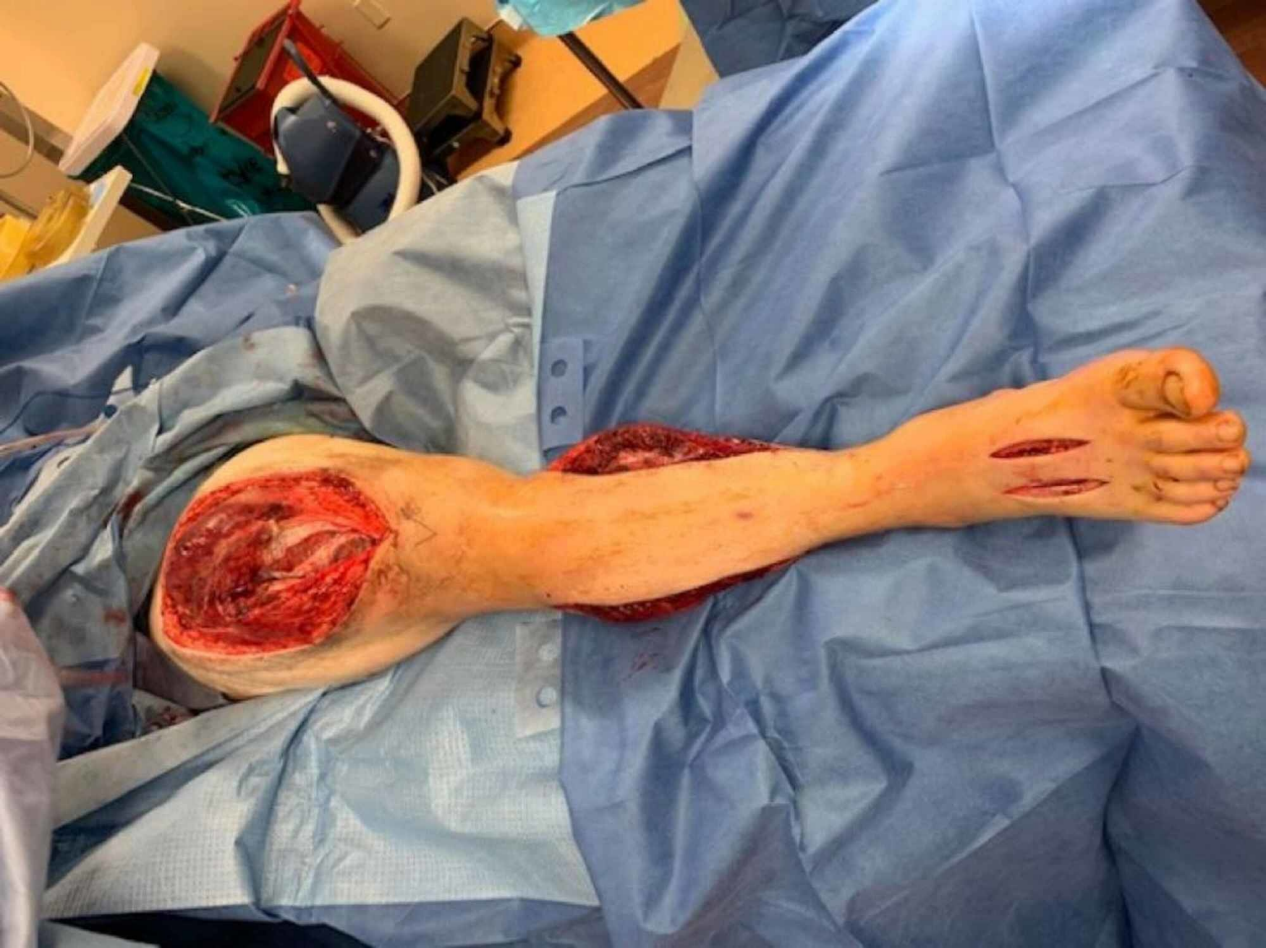
After foot fasciotomy, prior incision shows adequate thigh and leg fasciotomy

The patient had to be taken emergently to the operating room for a foot fasciotomy. On incising the fascia, an immediate bulge of the muscle was suggestive of foot compartment syndrome, and there was the quick return of pulse postoperatively (Figure 3).

Over the subsequent days, the patient's renal failure resolved, and once the swelling and edema on the limb had reduced, fasciotomy sites were closed with split skin thickness skin graft.

## Discussion

Compartment syndrome is a condition that can develop when there is an increase in interstitial pressure that builds up in the fascia compartment [3,4]. This pressure can cause tissue hypoxia leading to muscle damage [3]. Compartment syndrome is diagnosed based on physical examinations combined with elevated compartmental pressures [3,4]. These pressures, also known as the compartment delta pressure, are defined as the difference between the diastolic blood pressure and the intra-compartmental pressure [3]. Stryker® needle (Stryker Corporation, Kalamazoo, MI) can measure compartment pressure. A device that detects compartmental pressure by injecting saline solution into the compartment and measuring the resistance within the muscle [3,5,6]. The average pressure falls between 0 and 8 mmHg [3]. A value higher than 30 mmHg is indicative of compartment syndrome [5,6]. Clinicians use this as a cut-off value to need a fasciotomy [6]. The classic symptoms include pain, paresthesia, and pulselessness, this being a late presentation. Our patient presented with pain and paresthesia, and later when foot compartment syndrome manifested, he had also developed pulseless foot.

Opioid abuse can lead to long-term immobilization, which leads to crushing injuries resulting in compartment syndrome due to limb compression and muscle ischemia [6,7]. One reason is that in chronic use, a steady-state of the drug gets distributed and accumulated in the various body compartments [8]. The positioning of a limb against an immobile object may decrease local tissue perfusion and predispose to the development of acute compartment syndrome-like in our case [9]. Opioid-dependent patients require high doses of medications to manage their pain and often complain of pain to receive more drugs making it challenging to evaluate pain as a primary symptom [10]. In our patient on regular examination, it was difficult to assess if the patient was in pain due to surgery/drug-seeking behavior or the development of new compartment syndrome. Excessive opioid consumption or abuse long term can lead to various consequences, such as rhabdomyolysis, acute renal failure, and electrolyte disorders [8,10]. A severe complication of muscle damage can lead to rhabdomyolysis [11,12]. Muscle damage results in leakage of intracellular muscle toxins and proteins such as myoglobin, creatine phosphokinase (CPK), and electrolytes such as potassium into the bloodstream [11-13]. The classic triad of rhabdomyolysis is myalgia, weakness, and tea-colored urine due to myoglobin [11,12]. Symptoms range from being asymptomatic to life-threatening cardiac arrhythmias and acute renal failure [11,13]. Rhabdomyolysis causes renal vasoconstriction, intraluminal cast formation, and direct myoglobin deposition on the renal glomeruli [11-13].

Furthermore, alkalization of urine helps reduce the formation and precipitation of myo­globin casts in renal tubules [14]. This intravascular volume increases the glomerular filtration rate (GFR) and oxygen delivery and dilutes myoglobin and renal tubular toxins, reducing the risk for the development of acute renal failure [14]. The objective is alkalinizing the urine to a pH of greater than 6.5 and enhancing the flushing of casts from renal tubules by osmotic diuresis. Rhabdomyolysis and acute renal failure are treated with continuous venovenous hemofiltration (CVVH) if diuresis fails [15]. Studies have shown that extended dialysis with a high-flux, high-permeability membrane allowed effective elimination of myoglobin with a clearance of myoglobin that surpassed all previously reported dialysis techniques [16]. Our patient, due to extensive rhabdomyolysis, developed acute renal failure needing hemodialysis.

CPK is a protein that is released when muscle experiences trauma and is an important marker that we can use to evaluate muscle injury. Under physiologic circumstances, the plasma concentration of myoglobin is low (0 to 0.003 mg/dL). If more than 100 g of skeletal muscle is damaged, serum haptoglobin binding capacity becomes saturated [2]. Base levels of serum CPK in general populations vary from 35 to 175 U/L with ranges from 20 to 16,000 U/L, and this wide range depends on the type and duration of physical activity [15]. Attempts to correlate the elevation in CPK level with the severity of muscle damage and renal failure have had mixed results. However, significant muscle injury will have more creatine kinase elevations of more than >5,000 IU/L [13,14,17]. There is a direct correlation to the amount of muscle showing rhabdomyolysis to the elevation of CPK [14,17]. Our patient had extensive rhabdomyolysis and high CPK levels. Post fasciotomy CPK levels gradually dropped, but the CPK levels resulting from the development of foot compartment syndromes were not significant enough to show an upward trend. The failure of CPK to rise contributed to delayed recognition of foot compartment syndrome.

## Conclusions

Prolonged immobilization post drug abuse can lead to the development of compartment syndrome. Extensive muscle involvement with widespread rhabdomyolysis can lead to acute kidney injury needing renal replacement therapy. Biomarkers like CPK and regular examination can help unravel new compartments with compartment syndrome, which was not present in the initial presentation. Decreasing CPK levels may not reveal the presence of compartment syndrome in small muscle compartments and can be missed. It is essential not to miss the identification and management of additional compartment involvement in an urgent manner.

## References

[1] Elkbuli A, Sanchez C, Hai S, Mckenney M, Boneva D (2019). Gluteal compartment syndrome following alcohol intoxication: case report and literature review. Ann Med Surg.

[2] Sauret J, Marinides G, Wang G (2002). Rhabdomyolysis. Am Fam Physician.

[3] Giai Via A (2015). Acute compartment syndrome. Muscles Ligaments Tendons J.

[4] Osborn P, Schmidt A (2020). Management of acute compartment syndrome. J Am Acad Orthop Surg.

[5] Rademacher E, Miller P, Jordan E (2020). Management of fasciotomy incisions after acute compartment syndrome. J Pediatr Orthop.

[6] Duckworth A, McQueen M (2017). The diagnosis of acute compartment syndrome. JBJS Rev.

[7] Loloi J, Burton A, Walsh L, Sahu N, Jain R (2018). Bilateral compartment syndrome in intravenous drug abuse. Cureus.

[8] Mallappallil M, Sabu J, Friedman E, Salifu M (2017). What do we know about opioids and the kidney?. Int J Mol Sci.

[9] Halliwill J, Hewitt S, Joyner M, Warner M (1998). Effect of various lithotomy positions on lower-extremity blood pressure. Anesthesiology.

[10] Rao S, Mawn J, Lobaton G (2019). Opioid-related compartment syndrome and associated morbidity. Injury.

[11] Torres P, Helmstetter J, Kaye A, Kaye A (2015). Rhabdomyolysis: pathogenesis, diagnosis, and treatment. Ochsner J.

[12] Bagley W, Yang H, Shah K (2007). Rhabdomyolysis. Intern Emerg Med.

[13] Lane R, Phillips M (2003). Rhabdomyolysis. BMJ.

[14] Petejova N, Martinek A (2014). Acute kidney injury due to rhabdomyolysis and renal replacement therapy: a critical review. Crit Care.

[15] Sorrentino S, Kielstein J, Lukasz A (2011). High permeability dialysis membrane allows effective removal of myoglobin in acute kidney injury resulting from rhabdomyolysis. Crit Care Med.

[16] Huerta-Alardín AL, Varon J, Marik PE (2005). Bench-to-bedside review: rhabdomyolysis -- an overview for clinicians. Crit Care.

[17] Malinoski D, Slater M, Mullins R (2004). Crush injury and rhabdomyolysis. Crit Care Clin.

